# Always keep on learning

**DOI:** 10.4102/sajid.v37i2.370

**Published:** 2022-02-14

**Authors:** Harsha Lochan

**Affiliations:** 1 Department of Paediatrics and Child Health, Frere Hospital, East London, South Africa

My name is Harsha Lochan, and I work in East London, Eastern Cape, as an infectious diseases paediatrician. Becoming a paediatrician was a dream long before I entered medical school. I cannot really say that there was a particular moment when I decided that I wanted to be a doctor, but I have always wanted to make a difference somehow.

During my medical officer and registrar years, I was fortunate to be surrounded and educated by the most amazing paediatricians including Dr Helena Rabie, Dr Heloise Buys, Dr Louise Cooke and Prof. Brian Eley amongst others. I was in complete awe of all that they had achieved. Furthermore, improving children care at community level became important for me. This, together with learning and understanding the epidemiology of diseases, inspired me to pursue further training in infectious diseases. We learn by example; and I am fortunate that I was able to observe and learn from individuals who had passion, compassion and enthusiasm for their craft.

I completed my specialist and sub-specialist training in Cape Town, where patients were fortunate to have access to diagnostic, therapeutic and expert resources. When I travelled around the country whilst working for a non-governmental organisation (NGO), the disparities in healthcare resources between provinces swiftly became strikingly apparent. Most clinical staff in resource poor and constrained healthcare facilities were surviving day-to-day, and the very last thing they needed to hear was what else was wrong in an already poorly functioning system. The challenge therefore became ‘how do we fix this problem?’. From those primary health visits, I learned that what was urgently needed was to offer solutions to the challenges in the healthcare system and collaboratively work on ideas that would ultimately benefit the patient. Unfortunately, this is easier said than done. Change is not easy, and people can get in the way of change. One of the biggest challenges I faced was trying to influence changes in behaviour and approaches – I am probably not alone in this kind of effort. At first, my approach was to be assertive around clinical management approaches for common conditions and to encourage the guidelines that are relevant to paediatric infectious diseases are followed prudently. With time, I realised that this was not going to work. Genuine change needs to come from within the individuals, and by providing the relevant information, the process of change can be commenced. Picking one’s battle was an important lesson which I learnt in this process along with learning the value of persistence, perseverance, patience and repetition of information. This is an ongoing process, and I am constantly learning and evolving.

These experiences made me realise that I enjoy teaching and sharing information and resources – be it clinical, non-clinical, or research-related – with others. At times, my enthusiasm for the topic being taught was not matched with those of the students or even my colleagues, but I was still determined to share and teach.

With only two adult and two paediatric-trained specialists and a handful of pathologists – the infectious diseases (ID) community in the Eastern Cape province is small. The perception that ID only comprises management of patients with HIV and TB was difficult to break, and it was challenging to convince colleagues of the value of the knowledge and expertise that was on offer. For this reason, attempting to impart knowledge to junior and senior colleagues both at my hospital and on outreach visits to district level hospitals became a vital part of my job.

I would like to think that the doctors and nurses with whom I have interacted and shared my knowledge and experiences have gone on to give appropriate and improved care to their patients. Being able to teach is indeed a privilege for me; but I am also aware that I myself have so much still to learn.

My advice from my short career thus far is always to continue being a student and learn about new concepts and ideas – and not just in medicine. There certainly is no shortage of new medical and research information, and the past 2 years have shown how rapidly the world can change. I would also like to suggest that we challenge ourselves by engaging with new environments. There is always something to learn and something to offer when we try different things. It gives us a better understanding that not all societies are equal and that there is no ‘one size fits all’ principle.

Caring for children is indeed remarkable. It certainly keeps me on my toes and makes me always want to do my best. The innocence of a child is humbling and should be preserved for as long as possible.

## Data Availability

Data sharing is not applicable to this commentary.

